# The N276 Glycosylation Site Is Required for HIV-1 Neutralization by the CD4 Binding Site Specific HJ16 Monoclonal Antibody

**DOI:** 10.1371/journal.pone.0068863

**Published:** 2013-07-17

**Authors:** Sunita S. Balla-Jhagjhoorsingh, Davide Corti, Leo Heyndrickx, Elisabeth Willems, Katleen Vereecken, David Davis, Guido Vanham

**Affiliations:** 1 Institute of Tropical Medicine, Virology Unit, Antwerp, Belgium; 2 Institute for Research in Biomedicine, Bellinzona, Switzerland; 3 Biomedical Primate Research Centre, Rijswijk, the Netherlands; 4 Department of Biomedical Sciences, Faculty of Pharmacology, Veterinary and Biomedical Sciences, University of Antwerp, Antwerp, Belgium; University of Cape Town, South Africa

## Abstract

Immunogen design for HIV-1 vaccines could be based on epitope identification of naturally occurring neutralizing antibodies in infected patients. A tier 2 neutralizing monoclonal antibody (mAb), HJ16 recognizes a new epitope in the CD4 binding site (CD4bs) region that only partially overlaps with the b12 epitope. We aimed to identify the critical binding site by resistance induction in a sensitive primary CRF02_AG strain. In four independent dose-escalation studies, the N276D mutation was consistently the only alteration found and it was confirmed to be responsible for resistance to HJ16 by site-directed mutagenesis in envelopes (*envs*) of the homologous CRF02_AG, as well as of a subtype A and a subtype C primary isolate. This mutation removes an N-linked glycosylation site. The effect of N276D was very selective, as it failed to confer resistance to a range of other entry inhibitors. Remarkably, sensitivity to the CD4bs VRC01 and VRC03 mAbs was increased in the N276D mutated viruses. These data indicate that binding of the CD4bs specific HJ16 mAb critically depends on the interaction with the N276-glycan, thus indicating that HJ16 is the first glycan dependent CD4bs-specific mAb.

## Introduction

Designing a protective HIV-1 vaccine remains as much of a challenge today as in the 1980s. The envelope has been a main target in HIV-1 vaccine development since interaction of particular sites of HIV-1 envelope (Env) with cellular receptors is essential to mediate viral entry into human target cells. However, Env imposes a range of obstacles to vaccine development such as the presence of immunodominant non-neutralizing epitopes, high variability of neutralization-sensitive regions and their shielding by glycans. In addition, the flexibility of Env and its ability to undergo conformational changes during the process of cell entry severely limit the window of opportunity during which antibodies (Abs) can be effective [1].

Until recently, only a limited number of cross-neutralizing monoclonal Abs (mAbs) were characterized, including some that target the CD4 binding site (CD4bs), such as b12 and F105, but more recently a wealth of promising new very broadly neutralizing Abs were discovered e.g. Wu and Zhou described the CD4bs-specific VRC01 and VRC03 mAbs and Falkowska described PGV04. In addition, Walker et al. isolated PG9 & PG16 mAbs, recognizing, V1/V2 glycans, but also the PGT series, which was found to compete with the prototypical glycan binding 2G12 mAb [1]–[6].

We obtained a neutralizing mAb referred to as HJ16 from an African women in our HIV-1 cohort at the Institute of Tropical Medicine (ITM). It competed with CD4 for binding to gp120, but its neutralization spectrum was clearly different from b12, as it was mainly effective against tier 2 strains (which comprises the majority of primary HIV-1 isolates), whereas b12 rather neutralizes tier 1 strains [7], [8].

Our objective in the present study was to identify critical site(s) within the HJ16 epitope by resistance induction in a primary CRF02_AG strain that was very sensitive to HJ16 neutralization in a primary PBMC based neutralization assay.

## Materials and Methods

### Ethics Statement

This study was approved by the Institutional Review Board of ITM and the Ethical Committee of the University Hospital of Antwerp. All participants understood and signed an informed consent.

### Entry Inhibitors Used

The CD4bs specific HJ16 mAb was produced by one of the co-authors (Dr. Davide Corti). Other CD4bs targeting reagents include the broad neutralizing mAbs b12 and PVG04 obtained from Dr. Dennis Burton (Scripps Institute, La Jolla, CA USA), VRC01 and VRCO3 from Dr. John Mascola (Vaccine Research Centre NIH, Bethesda, MD USA), the llama heavy chain antibody (VHH) A12 from Dr. Theo Verrips (University of Utrecht, Utrecht, The Netherlands) [9], the CD4 miniprotein M48-U1 from Dr. Loic Martin (Centre à l’Energie Atomique, Saclay, France) [10] and soluble CD4 (sCD4), which was purchased from Progenics Pharmaceuticals, New York, USA. In addition we obtained the mAb 2F5 and 2G12 from Dr. Hermann Katinger (Polymun, Vienna, Austria) and the VHH 1B5 from Dr. Theo Verrips [11]. The CCR5 inhibitor Maraviroc was obtained from the NIH AIDS Research and Reference Reagent Program, Germantown, MD USA.

### Cells

Buffy coats from healthy donors were obtained from the Red Cross Blood Transfusion Center at the University Hospital of Antwerp and were used for isolation of peripheral blood mononuclear cells (PBMC) by LymfoPrep (Axis-Shield, Oslo, Norway). These PBMC were adjusted to 1×10^6^/ml in complete culture medium, consisting of RPMI 1640, 15% fetal calf serum (FCS), 0.03% L-glutamine and 50 µg/ml gentamycin (Lonza, Verviers, Belgium) and 2 µg/ml polybrene (Sigma-Aldrich, Bornem, Belgium). Cells were stimulated with 0.5 µg/ml phytohemagglutinin (PHA, Oxoid, Hampshire, UK) for 2 days and 1 day with 200 U/ml interleukin-2 (IL-2, Gentaur, Brussels, Belgium) in a 7% CO_2_ incubator at 37°C and then used for neutralization assays.

The following cell lines were obtained from the NIH AIDS Research and Reference Reagent Program: TZMbl (cat no. 8129); HOS.CD4-R5 (cat no. 3318) and GHOST(3)X4/R5 (cat no. 3943). The HEK 293T cells (CRL-11268) were obtained from the American Type Culture Collection (ATCC, Manassas, Virginia, USA).

### Replication Competent HIV-1 Infectious Virus (IV) and Single-cycle HIV-1 Pseudoviruses (PV)

The VI1090 CRF02_AG strain was isolated in a classical PBMC culture from a 38 year old heterosexual Nigerian man. The *env* gene was amplified from PBMC cultures followed by cloning into the pcDNA4/TO expression vector [12]. Additional PV constructs, derived from a primary clade C virus VI829 [13] and from a near full length subtype A clone 92RW009.6 (NIH4006) [14], were also included. PV were generated in a 24-well plate by transfection of HEK 293T cells with pNL4-3.LucR^−^E^−^, obtained from NIH AIDS Research and Reference reagent program and the *env* containing plasmid, as previously described [12]. Sequencing of the PV constructs and phylogenetic analysis of the complete gp160 confirmed identity between the *envs* of the pseudoviruses and the original replicating viruses VI1090, VI829 and 92RW009.6 respectively. The full length *env* sequence of the VI1090 PV construct has been deposited with GenBank (accession number HQ912710).

### Mutagenesis

Site-directed mutagenesis was carried out on the PV constructs (VI1090, VI829 and 92RW009.6) using the QuikChange Lightning Site-Directed Mutagenesis kit (Stratagene, La Jolla, CA, USA) following the guidelines of the manufacturer. Primers used to introduce the desired mutation were: VI1090_276D_Fwd (5′-G GTA GTG ATT AGA TCT GAA **GAT** ATC ACA AAC AAT GCC AAA -3′) and VI1090_276D_Rev (5′-TTT GGC ATT GTT TGT GAT ATC **TTC** AGA TCT AAT CAC TAC C -3′); VI829_276D_Fwd (5′-GAG ATA ATA ATT AGA TCT GAA **GAT** ATA ACA GAT AAT GTC AAA AC -3′) and VI829_276D_Rev (5′-GT TTT GAC ATT ATC TGT TAT **ATC** TTC AGA TCT AAT TAT TAT CTC -3′); 92RW009_276D_Fwd (5′-G ATA ATA ATT AGA TCT GAA **GAT** ATT ACA AAC AAT GCC AAA ACC -3′) and 92RW009_276D_Rev (5′-GGT TTT GGC ATT GTT TGT AAT **ATC** TTC AGA TCT AAT TAT TAT C 3′). Underlined and in bold the mutated N276 D. The presence of the mutation was confirmed by sequencing the complete *env* gp160.

### Neutralization Assays

*Extended incubation PBMC-based assay*: This assay has been previously described [15], [16]. Briefly, the virus stock was diluted in a fivefold series from 1/2 to 1/6250 in culture medium (RPMI-1640 medium supplemented with 15% FCS and 200 U/ml IL-2). Ninety µl of each virus dilution were mixed with 5 µl of plasma +5 µl of culture medium (final plasma concentration 1/20) or 5 µg mAb +5 µl IgG-depleted HIV-negative plasma (IgG was removed with a Protein G column [17]) to give a final mAb concentration of 50 µg/ml. As a negative control, either HIV seronegative plasma (pooled from 100 healthy donors) or purified IgG thereof were used for incubation with the virus dilution. After an incubation phase of 24 hours, 20 µl of each plasma/virus or mAb/virus mixture were first dispensed in quadruplicate into flat bottom 96-well microplates and 75,000 PBMC in 100 µl culture medium were added to each well. Plates were then left in a CO_2_ incubator at 37°C during the absorption phase of 1 hour. Afterwards, cells were washed three times by centrifugation at 2000 rpm for 10 minutes, the supernatant was aspirated and 180 µl fresh culture medium was added to each well. After 14 days of incubation in a CO_2_ incubator, 200 µl of the supernatant were mixed with 50 µl Nonidet P40 (0.25% in PBS; Fluka, Sigma-Aldrich, Puurs, Belgium) to disrupt virions and this mixture was analyzed in our in house HIV p24 antigen assay [17]. Wells with an OD>0.3 were considered to be positive.

Virus titers were calculated by the method of Reed and Muench [18]. Neutralization activities are presented as the percentage reduction in infectious titer following incubation with plasma from the patient or mAb relative to its titer following incubation with control HIV seronegative pooled plasma or purified IgG from this pool respectively. An 80% reduction in titer was considered significant. Neutralization indices were translated into % neutralization by using the formula: 100-POWER(10;2-neutralization index). A detailed protocol can be found at http://www.europrise.org/documents/NEUTNET/SOPS/6B_ITG_PBMC.pdf.

*Cell line based assays*: Neutralization capacity of HJ patient plasma and HJ16 mAb was measured against a fixed dose of infectious virus (IV) or pseudotyped virus (PV) on TZMbl, whereas PV only was used in HOS cells and the related GHOST cells [19], [20]. After 48 h of incubating cells, virus and plasma or Ab, luciferase reporter gene production was quantified upon cell lysis and addition of firefly luciferase substrate (Perkin-Elmer, Waltham, Massachusetts USA). Emitted relative light units (RLUs) were quantified on a LB 941 Berthold® luminometer (Bad Wildbad, Germany). SteadyLite® was used as a substrate in the TZMbl assay and BriteLite® in the GHOST and HOS assay (both are from Perkin-Elmer). The fixed dose of virus to be used was determined in a preliminary titration experiment, wherein 1×10^4^ TZMbl, HOS or GHOST cells were infected with a range of viral dilutions in a total volume of 200 µl to establish the dose, which resulted in a signal of 50,000 to 100,000 RLU. Background RLUs (only cells, no virus) for TZMbl, HOS and GHOST were ∼4,000, 40 and 50 respectively. In the TZMbl assay diethylaminoethyl-dextran (DEAE-dextran, Sigma, Belgium) was used at 15 µg/ml final concentration to enhance virus infectivity. In the actual neutralization experiments, Abs or plasma were pre-incubated with pseudoviruses for 1 h at 37°C. The Ab concentration or plasma dilution causing a 50% reduction in luciferase reporter gene production was determined by linear regression analysis in Microsoft Office Excel as described on http://www.hiv.lanl.gov/content/nab-reference-strains/html/Protocol-for-Neutralizing-Antibody-Screening-Assay-for-HIV-1-in-TZM-bl-Cells-November-2010.pdf. The HIV-seronegative plasma pool and purified IgG, already described in the PBMC assay, were used as negative controls in cell line based assays.

### Structural Analyses

The crystal structure coordinates of the different gp120 molecules interacting with sCD4 (pdb 1gc1) or the human CD4bs-specific antibodies PGV04 (pdb 3se9), b12 (pdb 3dnl) and VRC01 (pdb 3ngb) were used to identify the footprint of interaction of each ligand on gp120 within an interaction distance of 5 A°. The solved gp120-bound glycans and their atomic coordinates were highlighted as described in the deposited pdb file. The details of hydrogen bonds between Tyr-28 and Ser-30 of the VRC01 light chain and the protein-proximal N276-acetylglucosamine (as described in [3]) were determined as polar contacts between the two light chain residues and the neighboring residues. Figures were made with Mac PyMOL.

## Results

### Neutralization Breadth of HJ16 and Selection of a Sensitive Isolate to Induce Resistance

The HJ16 mAb was isolated from memory B cells of an HIV-1 subtype C infected Congolese woman (identified HJ), whose plasma showed an exceptionally broad neutralizing profile (neutralizing 15 out of 20 strains tested) upon screening in the “extended incubation” PBMC assay (Table 1). In the same assay, the HJ16 mAb neutralized six strains. Of these 4 were also neutralized by the plasma but two strains (92RW009.6 and VI1380) were not, indicating that the neutralizing capacity of the plasma is not recapitulated by this single mAb. Clearly, the CRF02_AG strain VI1090 was very susceptible to neutralization by both the original plasma and the HJ16 mAb.

**Table 1:** Neutralization profile of HJ patient plasma and HJ16 mAb in the 24 h/1 h/14d^a^ extended incubation PBMC assay.

Subtype	Strain	HJ plasma	HJ16 mAb
		1/20 dilution^b^	50 ug/ml^b^
A	VI191	**96** c	66
	92RW009.6	E^c^	**96**
	CA1	**99**	E
B	SF162	**99**	0
	MN	E	E
	BaL	**98**	66
	89.6	**91**	55
C	VI829	**86**	**91**
	VI882	**99**	E
	VI1358	**95**	41
	92Br025	**98**	78
D	VI824	78	55
	CI13	**99**	**80**
CRF01_AE	VI1888	**89**	22
CRF02_AG	VI1090	**95**	**99**
	VI2680	0,0	37
	CI20	**93**	71
	CA18	**97**	**80**
	VI1380	0,0	**95**
	VI2727	**84**	E

a In this assay virus and plasma or mAb are incubated during 24 hours, followed by an absorption time of 1 hour during which the mAb/virus mixture is co-incubated with donor PBMC. After washing the PBMC cultures are maintained for 14 days.

b % Neutralization obtained with 1∶20 plasma dilution or 50 µg/ml mAb during the incubation phase.

c ≥80% reduction in virus titer is highlighted, E = enhancement of infection.

In a previous study we have shown that the neutralization capacity of patient sera, as well as that of individual mAbs is highly dependent on the type of assay used [8]. Exploring the activity of HJ16 against VI1090 in other types of neutralization assays using PBMC or cell lines as target cells, in combination with primary replicating infectious virus (IV) or non-replicating pseudovirus (PV) confirmed sensitivity of VI1090 to 50 µg/ml HJ16 in all cases, whereas a 1/20 dilution of the corresponding HJ plasma showed a weaker activity (Table 2). A further titration indicated that 0.15 µg/ml of HJ16 neutralized VI1090 at a 50% level in the TZMbl assay.

**Table 2:** Neutralization profile of HJ patient plasma and HJ16 mAb against VI1090 virus in various assays.

	PBMC based^a^	Cell line based^b^			
		TZMbl_IV	TZMbl_PV	GHOST_PV	HOS_PV
HJ plasma^c^	**95** e	42^f^	48^f^	43^f^	**61** f
HJ16 mAb^d^	**99** e	**97** f	**100** f	**100** f	**100** f

a The extended incubation PBMC assay was performed with the infectious virus VI1090.

b Both infectious virus (IV) and pseudovirus (PV) were used in the TZMbl assay, whereas only PV was used in the GHOST and HOS assays.

c HJ plasma used at 1∶20.

d mAb used at 50 µg/ml.

e % Reduction in virus titer.

f % Reduction in luciferase activity.

In conclusion, the VI1090 virus is very sensitive to neutralization by HJ16, irrespective the assay used and was therefore selected for resistance induction.

### Resistance Induction in VI1090 with HJ16 Results in a Unique N276D Mutation

The primary VI1090 strain was cultured in activated donor PBMC in the presence of increasing concentrations of HJ16, starting at 0.10 µg/ml ( = just below the IC50 in TZMbl). After each week, p24 ELISA was performed and positive cultures were passaged again with a 2–5 fold increased concentration of HJ16. After 35 days, we obtained a virus growing in the presence of 187.5 µg/ml HJ16 (∼2,000 fold increase). This procedure was reproduced in four independent experiments. Table 3 shows that in each case a similar high-level resistance against HJ16 was obtained, whereas the sensitivity towards TriMab remained unchanged. TriMab is a combination of 2F5 (targeting the membrane proximal part of gp41), 2G12 (recognizing a specific configuration of glycans at positions N295, N332, N339, N386 and N392 on gp120) and b12 (prototypical CD4bs mAb) [1].

**Table 3:** Neutralization sensitivity to HJ16 and TriMab of VI1090 before and after HJ16 resistance induction.

	HJ16	TriMab^a^
VI1090_WT^b^	0.20^c^	11.75
	0.57	32.01
	0.69	27.04
VI1090_sens 1^b^	0.53	33.63
VI1090_sens 2	1.79	34.02
VI1090_sens 3	1.80	32.97
VI1090_sens 4	0.70	12.48
VI1090_res_1^b^	>200	25.59
VI1090_res_2	>200	9.93
VI1090_res_3	>200	11.93
VI1090_res_4	>200	10.51

a TriMab consists of b12, 2G12 and 2F5.

b VI1090_WT is the original HJ16 sensitive stock used to initiate resistance induction; VI1090_sens is the WT cultured in parallel to the resistance induction but without HJ16; VI1090_res is the WT cultured in the presence of HJ16 up to a final concentration of 187.5 µg/ml.

c IC_50_ values (µg/ml) in TZMbl assay.

Sequencing revealed the presence of a single point mutation in all four HJ16 resistant variants whereby the neutral asparagine (N) was replaced by the negatively charged aspartic acid (D) at position 276 (numbering according to HxB2), resulting in the loss of a potential N linked glycosylation site (PNGS) [21]. Searching the Los Alamos database indicated that this N276D point mutation is only seen in 0.85% (19/2247) of group M isolates. Sequences of the four HJ16 resistant VI1090 viruses as well as the control viruses, grown in absence of HJ16, have been deposited at GenBank (accession numbers KC980915-KC980920).

### The N276D Mutations Confers Selective Resistance to HJ16 across Subtypes

In order to ascertain that this rare mutation was indeed responsible for resistance to HJ16, N276D site-directed mutants were generated in the *envs* of three sensitive strains from different subtypes, selected from Table 1: the original VI1090 (CRF02_AG), 92RW009.6 (subtype A) and VI829 (subtype C). Next, the impact of N276D on the sensitivity to HJ16 and other CD4bs mAbs (b12, VRC01 and VRC03) [1], two llama single heavy chain antibodies or VHHs (A12 and 1B5) [9], [11]; the CD4 “miniprotein” M48-U1 [10] and soluble CD4 was assessed in the TZMbl neutralization assay. In addition, the TriMab combination and the CCR5 inhibitor Maraviroc were used.

Clearly N276D mutation produced high-level resistance specifically to HJ16 in all three isolates (Table 4). There was no evidence of cross-resistance of the N276D mutants to other entry inhibitors used, as the difference in IC_50_ of mutant/WT was always less than twofold. Remarkably the mutation, N276D conferred a 3 to 13 fold increase of sensitivity to both VRC01 and VRC03. The exception is VI829 Env containing PV, which is not sensitive to VRC03 both in WT and mutant form.

**Table 4:** Influence of N276D in different Envs on their sensitivity to various entry inhibitors in TZMbl assay.

Assay	VI1090^a^		92RW009.6^a^		VI829^a^	
	WT	N276D	WT	N276D	WT	N276D
**HJ16**	0.085^b^	>200	0.123	>200	48.17	>200
b12	>200	>200	>200	>200	9.68	4.20
A12	>100	>100	>100	>100	>100	>100
1B5	>100	>100	28.71	44.06	6.65	4.57
M48-U1	0.16	0.16	0.50	0.59	0.04	0.02
sCD4	13.81	7.11	39.56	29.60	0.55	0.34
VRC01	0.16	0.03	0.44	0.14	0.90	0.07
VRC03	0.04	<0.01	2.69	0.3	>50	>50
Maraviroc	<0.01	<0.01	<0.01	<0.01	<0.01	<0.01
TriMab	5.76	12.56	16.53	27.65	60.20	28.71

a The Envs from VI1090 (CRF02_AG), 92RW009.6 (subtype A) and VI829 (subtype C) were either used as such (WT) or after site-directed mutagenesis at the 276 position to construct PV for the TZMbl assay.

b Mean IC_50_ (µg/ml) of at least two (VRC01 and VRC03) or three (all others) independent experiments are shown.

### Structural Analysis Reveals an Interaction between VRC01 Light Chain and N276-linked Glycan

**Figure 1:**
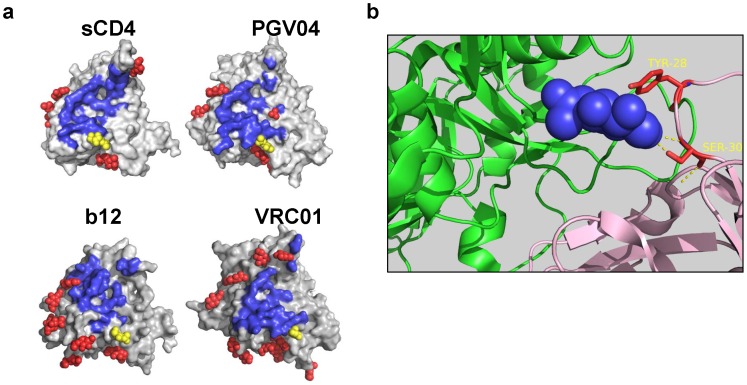
Positioning in yellow of N276-bound glycans (yellow spheres) in solved structures for sCD4, PGV04, b12 and VRC01. The blue highlight indicate the footprint of the cognate ligand and the red spheres the other glycans. A N276 glycan on gp120 structures. In blue are shown the footprints of CD4 (pdb accession n° 1gc1) and the CD4bs-specific antibodies PGV04 (pdb n°: 3se9), b12 (pdb n°: 3dnl), and VRC01 (pdb n°: 3ngb). In each structure the N276-bound glycan is highlighted in yellow, other, surrounding glycans in red.** B.** Detail of the molecular interaction between the residues tyrosine 28 and threonine 30 of the VRC01 light chain with N-acetyl-glucosamine of the N276-bound glycan (3ngb). Figures were made with PyMOL.

The presence of a glycan at position 276 was confirmed in several gp120 structures including as ligands sCD4, PGV04, b12 and VRC01 (Figure 1a) and shown to be proximal to the CD4bs on gp120. In the case of VRC01 the structural analysis revealed that VRC01 interacts with the N-acetyl-glucosamine of the N276-linked glycan through the light chain residues tyrosine 28 and threonine 30 (Figure 1b). These data show that VRC01 interaction with gp120 may also involve the recognition of a glycan.

## Discussion

Previous analysis, comparing b12 and HJ16 indicated that these mAb recognize a related, but not identical part of the CD4bs: the binding of gp120 to solid-phase CD4 was inhibited by both mAb to a similar extent, but cross-competition between b12 and HJ16 for binding to gp120 showed incomplete heterologous inhibition. Moreover, the D368R mutation in the heart of the CD4bs, well known to abrogate binding of b12, didn’t affect HJ16 binding to gp120. The neutralization spectrum of both mAb was clearly different and largely complementary: while b12 neutralized most tier 1 viruses, HJ16 failed against these “easy-to-neutralize” viruses, such as BaL, SF162 and MN, but was active against many tier 2 viruses. Unfortunately, we failed to characterize the epitope, using overlapping peptides, which suggested that HJ16 recognizes a discontinuous (conformational) epitope [7], [8].

In order to uncover important attachment sites for HJ16 binding on gp120, we used resistance induction in the CRF02_AG strain VI1090, shown to be susceptible to HJ16 in multiple neutralization assays [8]. In all four resistance induction experiments the rare N276D point mutation emerged. The area around 276 includes also N262 and N289 and therefore has been reported as a possible glycosylation site [21]. We applied site directed mutagenesis in the VI1090 CFR02_AG *env* to confirm that this mutation was responsible for the resistance to HJ16 in VI1090 and showed in addition that introducing the N276D mutation in sensitive A and C isolates also induced full resistance to HJ16. These results could be explained by the fact that the 276 position is located in the C2 part of the outer domain of gp120, which in the three-dimensional structure is relatively close to, but yet just outside the CD4bs [22]. The mutation failed to clearly alter the sensitivity towards most CD4bs targeting compounds, such as sCD4, M48-U1 (CD4 miniprotein), CD4bs mAb b12 and the VHH A12, but intriguingly the sensitivity towards the novel VRC01 and VRC03 was clearly increased. As could be expected, the mutation didn’t affect the activity of Maraviroc (CCR5 inhibitor), 1B5 (targeting the CD4i site – submitted for publication) or TriMab, consisting of 2G12 (recognizing a specific configuration of glycans, but not including N276, on gp120) [23], [24], 2F5 (recognizing the MPER in gp41) and b12 [1]. Together with our previous findings, the present observations confirm the uniqueness of this HJ16 mAb that probably blocks the gp120-CD4 interaction by binding a glycan near the CD4bs.

The importance of glycosylation in binding and activity of HIV-neutralizing antibodies has recently gained interest. In Lavine’s study, mutations of subtype B JR-FL and YU-2 viruses at position 234 and 241 (of the inner gp120 domain) as well as 332 and 386 (outer gp120 domain) decreased sensitivity to at least 2 out of 9 broadly neutralizing patient sera. Of these N332S and N386T also abrogated the neutralizing capacity of mAb 2G12, as could be expected. Conversely, a number of PNGS in V1 (position 160), V2 (197), V3 (301) and gp41 (616) increased sensitivity to neutralization by 5 to 9 out of the 9 patient sera. Remarkably the N276S mutation also increased sensitivity to neutralization by two patient sera [21]. A second paper by Wang et al. studied the effect of glycan removal on sensitivity to various neutralizing mAbs and found that several mutation of PNGS in V4/V5 and C2/C3/C4 regions of gp120 from a Chinese BC strain alter neutralization sensitivity of these mAbs to a variable extent, but consistently reduced sensitivity to the glycan specific PG16 [22]. Unfortunately mutation of 276 was not studied by Wang.

A few years ago, Walker et al. described a series of very broadly neutralizing PGT mAbs, which recognize the glycans at position N332 in the C3 region of gp120 [4]. Moore et al. showed that mutation of N332 abrogates the neutralizing activity of mAb PGT128 [25], whereas Moldt et al found that the related glycan-specific mAb PGT121 can protect against infection with SHIVSF162 [26]. All these findings underline the importance of PNGS Abs for future vaccine development.

Conversely, the paper by McGuire at al. (which appeared during the revision of this manuscript) shows enhanced binding of both the mature and germline reverted version of the potent CD4bs-specific VRC01 to the 276D mutant subtype C virus (labeled 266c), which lacks the natural complex glycan as compared to WT N276 [27]. Available structural data, however, showed an interaction between VRC01 light chain and the N276-bound N-acetyl glucosamine. It is important to note that the gp120 used to make co-crystals are treated with glycosidases (Endo H in the case of the VRC01 structure which leaves a N-acetyl glucosamine residue on N276) and that the glycan bound to N276 is most likely of larger size and complexity on the native gp120. The presence of a bulkier glycan at N276 position may indeed clash with VRC01 light chain, as confirmed by Jardine et al [28], thus reducing its overall affinity for the N276 glycosylated gp120 as compared to gp120 lacking this glycan, as already suggested by McGuire’s data. Our data on increased sensitivity of N276D mutants of subtype A, C and CRF02 Env towards neutralization by VRC01 therefore provide an important functional correlate to McGuire’s and Jardine’s observations and indicate that the interactions between the N276 glycan and the VRC01 light chain residues is in fact not a requirement for antibody binding.

In conclusion, similar to PG9 and PGT128, binding outside the CD4bs [22], [25] and in contrast to other CD4bs mAb, such as VRC01, the HJ16 epitope may be constituted of a glyco-peptide. Whereas McGuire’s data, supplemented with ours on VRC01 suggest that the N276 glycosylation (present in most WT HIV-1) hinders the recognition and maturation of VRC01-like antibodies *in vivo*, this glycan is most probably required for HJ16-like antibody induction. This knowledge is of obvious interest to design new Env-specific immunogens for the induction of ‘tier 2-type’ CD4bs specific neutralizing antibodies.
